# Circulating fibroblast growth factor 21 is associated with blood pressure in the Chinese population: a community-based study

**DOI:** 10.1080/07853890.2025.2500689

**Published:** 2025-05-12

**Authors:** Juanying Zhen, Shuyun Liu, Lin Liu, Xiaodan Zheng, Guoru Zhao, Jianguo Liang, Aimin Xu, Chao Li, Lijie Ren, Jun Wu, Bernard Man Yung Cheung

**Affiliations:** ^a^ Department of Medicine, School of Clinical Medicine, The University of Hong Kong, Queen Mary Hospital, Pokfulam, Hong Kong SAR, China; ^b^ Department of Neurology, Peking University Shenzhen Hospital, Shenzhen, China; ^c^ Department of Neurology, Shenzhen Longhua District Central Hospital, Shenzhen, China; ^d^ CAS Key Laboratory of Human-Machine Intelligence-Synergy Systems, Research Center for Neural Engineering, Shenzhen Institute of Advanced Technology, Chinese Academy of Sciences, Shenzhen, China; ^e^ ’Precision Health Research Center Company Limited, Hong Kong SAR, China; ^f^ State Key Laboratory of Pharmaceutical Biotechnology, The University of Hong Kong, Pokfulam, Hong Kong SAR, China; ^g^ Department of Neurology, The First Affiliated Hospital of Shenzhen University, Shenzhen Second People’s Hospital, Shenzhen, China; ^h^ Institute of Cardiovascular Science and Medicine, The University of Hong Kong, Pokfulam, Hong Kong SAR, China

**Keywords:** Fibroblast growth factor 21, biomarker, blood pressure, hypertension

## Abstract

**Background:**

Our research team previously found that fibroblast growth factor (FGF) 21, a circulating hormone, was significantly associated with atherosclerosis in human and animal models. The relationship between FGF21 and blood pressure (BP) is rarely studied in the Asian population. Therefore, we aimed to explore the relationship of FGF21 with BP in a Chinese population.

**Methods:**

We analysed data on 1051 participants from the Shenzhen–Hong Kong United Network on Cardiovascular Disease (SHUN-CVD) study.

**Results:**

The medians of FGF21 level were 355.1 pg/mL (IQR 234.3–574.8 pg/mL) for hypertensive patients and 253.5 (IQR 136.9–403.3 pg/mL) for non-hypertensive participants. Ln-transformed FGF21 level was associated with both systolic and diastolic BP (systolic BP: *B* = 4.45 [95% CI 3.41–5.49]; *p* < .001; diastolic BP: *B* = 2.72 [95% CI 2.03–3.42]; *p* < .001). After adjusting for sex, age, body mass index, hypercholesterolaemia, diabetes, alcohol consumption, smoking and physical activity, the association remained significant (systolic BP: *B* = 1.99 [95% CI 1.01–2.97]; *p* < .001; diastolic BP: *B* = 1.36 [95% CI 0.69–2.04]; *p* < .001). Serum FGF21 level was associated with hypertension (quartile 4 vs. quartile 1, OR = 4.19 [95% CI 2.65–6.61]; *p* for trend < .001).

**Conclusions:**

This is the first study to elucidate the relationship of FGF21 with BP in the Asian population. FGF21 is significantly associated with BP. Besides its use as a biomarker, FGF21 may be a new drug target for hypertension treatment.

## Introduction

Fibroblast growth factor (FGF) 21 is a 181-amino acid peptide hormone and a member of the FGF superfamily. FGF21 could be dramatically stimulated in fasting and starvation status. Human FGF21 is secreted by the liver, adipose tissues, skeletal muscle and pancreas. Due to the absence of heparin-binding domain, it could be secreted into the circulation and act as a circulating hormone [1]. FGF21 has been reported to be a regulator of glucose and lipid homeostasis [2]. In obese rodents and diabetic primates, administration of FGF21 resulted in beneficial metabolic effects including improved insulin sensitivity, significant weight reduction, and a decrease in both blood triglyceride and low-density lipoprotein cholesterol [3,4].

Despite its biological effects on glucose and lipid metabolism, circulating FGF21 level is increased in participants with adverse lipid profiles, obesity and impaired glucose tolerance [5–7]. In the Fenofibrate Intervention and Event Lowering in Diabetes Study including 9697 participants, plasma FGF21 level was significantly associated with cardiovascular outcomes after a 5-year follow-up period [8]. Given the difference in the findings between animal and human studies, the hypothesis of FGF21 resistance, which is analogous to insulin resistance, was formulated. A recent Mendelian randomization study has found a causal relationship between FGF21 and improved lipid profile, which supported the existence of FGF21 resistance in humans [9].

Adverse lipid profile is associated with high blood pressure (BP) and the development of hypertension [10]. The association between FGF21 and hypertension was found in US adults. However, there are discrepancies between Western and Eastern populations in circulating FGF21 and the prevalence of hypertension [11–13]. The relationship between FGF21 and hypertension in the Asian population is unclear and therefore further investigation is needed. Our research team previously revealed that FGF21 was associated with type 2 diabetes (T2D) and carotid atherosclerosis based on the data from Hong Kong Cardiovascular Risk Factor Prevalence Study (CRISPS) [14,15]. In our 5.4-year prospective study, elevated FGF21 level was found in participants with prediabetes and diabetes at baseline. FGF21 was a strong predictor for diabetes development [14]. We demonstrated that FGF21 was significantly associated with carotid atherosclerosis. The association was independent of established cardiovascular risk factors including adverse lipid profiles and C-reactive protein [15]. Our preclinical study has found that FGF21 prevented atherosclerosis by suppressing hepatic sterol regulatory element-binding protein-2 and inducting adiponectin [16]. Therefore, in this study, we aimed to explore the relationship of FGF21 with BP and hypertension in a Chinese population.

## Methods

### Study participants

Shenzhen–Hong Kong United Network on Cardiovascular Disease (SHUN-CVD) is a population-based study conducted since 2020 in Shenzhen, China. By the end of 2022, a total of 3400 participants above 18 years old have been continuously recruited from communities to establish a comprehensive database of cardiovascular risk factors in a Chinese population. Interview, physical examination and laboratory tests are the three main parts of the survey. Demographic data, lifestyle behaviours, medical condition, drug usage, family history and body measurement were collected during the recruitment. Data on biomarker levels were available from 1060 participants who were recruited in September 2021. In the present study, 1051 people with complete data on circulating FGF21 level, BP, hypertension and relevant variables were included.

The protocols of this study were approved by the institutional review boards of both Peking University Shenzhen Hospital and the University of Hong Kong. Written informed consent was obtained from all study participants.

### FGF21 measurement

Blood samples were collected from the participants after an eight-hour fasting by nurses. Serum samples were processed immediately and stored at −80 °C until assayed. FGF21 was measured using ELISA kits (Antibody and Immunoassay Services, University of Hong Kong, Pokfulam, Hong Kong SAR, China). Serum FGF21 level has been confirmed to be stable after one to six freeze–thaw cycles, with the coefficients of variation (CVs) being 8.1% [17]. Briefly, serum sample was diluted with assay buffer and analysed together with quality controls following the instructions of the manufacturer. The average intra- and inter-assay CVs were 4.5% and 6.9%, respectively. Detailed procedures can be found on the website of ImmunoDiagnostics Limited, University of Hong Kong [18]. Laboratory technicians were blinded to the study participants’ clinical characteristics.

### Variables of interest

BP was measured in the right arm of participants by physical examiners following standard procedures. Participants were required to have at least 15 min of rest before the measurement. BP was recorded three times using a mercury sphygmomanometer. Participants who answered yes to the question ‘Have you ever been diagnosed with hypertension by a doctor’ were considered to have hypertension diagnosis. Hypertension was defined as one of the following criteria: (1) hypertension diagnosis, (2) taking medication for hypertension, (3) average systolic BP ≥140 mmHg or (4) average diastolic BP ≥90 mmHg.

Participants who had any tobacco product consumption daily or occasionally, or a history of smoking were considered as smokers. Participants who consumed any type of alcoholic beverage (e.g. beer, wine and liquor) at least once a week were considered as drinkers. Participants who took part in 30 or more minutes of moderate or vigorous activity three days per week were considered to be physically active. Body mass index (BMI) was calculated as participants’ weight (in kg) divided by the square of height (in m^2^). Hypercholesterolaemia was defined as hypercholesterolaemia diagnosis, total serum cholesterol level ≥5.2 mmol/L (200 mg/dL) or taking medication for hypercholesterolaemia. Diabetes was defined as diabetes diagnosis, fasting plasma glucose level ≥7.0 mmol/L (126 mg/dL), haemoglobin A1c level ≥6.5% or taking diabetic medication.

### Statistical analysis

Clinical characteristics of the participants were analysed using Chi-squared test for categorical variables and one-way analysis of variance for continuous variables. Participants were classified according to hypertension status in Table 1 and quartiles of serum FGF21 level in Table 2 (quartile 1: <156.6 pg/mL, quartile 2: 156.6–271.8 pg/mL, quartile 3: 271.9–429.1 pg/mL and quartile 4: ≥429.2 pg/mL). The distribution of FGF21 level was skewed, so we applied a natural logarithmic transformation to the data before analysis. We investigated the association of FGF21 level with systolic and diastolic BP using linear regression. The association between FGF21 quartiles and hypertension was investigated using logistic regression. Sex, age, BMI, anti-hypertensive treatment, hypercholesterolaemia, diabetes, alcohol consumption, smoking and physical activity were considered as confounders in multivariable-adjusted models. We also conducted two sensitivity analyses to test the robustness of our results. The first analysis excluded participants who were taking anti-hypertensive medication, and the second analysis incorporated the estimated glomerular filtration rate (eGFR) calculated by cystatin C [19] into the multivariable-adjusted models. Furthermore, we examined the association between ln-transformed FGF21 levels and the odds ratio for hypertension by using restricted cubic splines with four knots at percentiles of 5th, 35th, 65th and 95th [20].

**Table 1.** Clinical characteristics of all 1051 participants among participants with and without hypertension.

	No hypertension	Hypertension	*p* Value
*N*	807	244	
Age, years	44.0 ± 10.1	49.5 ± 9.4	<.001
Men, %	144 (59.0)	336 (41.6)	<.001
BMI, kg/m^2^	23.2 ± 3.4	25.2 ± 3.7	<.001
SBP, mmHg^a^	113.7 ± 11.7	137.2 ± 13.7	<.001
DBP, mmHg^a^	75.1 ± 7.9	90.4 ± 9.5	<.001
Smoking, %	151 (18.7)	67 (27.5)	.004
Physical activity, %	364 (45.1)	125 (51.2)	.108
Alcohol consumption, %	50 (6.2)	23 (9.4)	.111
Hypercholesterolaemia, %	285 (35.3)	99 (40.6)	.156
Diabetes, %	55 (6.8)	29 (11.9)	.015
Glucose, mmol/L	4.61 ± 1.19	4.83 ± 0.96	.008
Total cholesterol, mmol/L	4.90 ± 0.89	4.98 ± 0.89	.213
Triglycerides, mmol/L	1.43 ± 1.53	1.76 ± 1.16	.002
HDL, mmol/L	1.40 ± 0.30	1.31 ± 0.27	<.001
LDL, mmol/L	2.71 ± 0.75	2.77 ± 0.69	.281
FGF21, pg/mL	253.5 (136.9–403.3)	355.1 (234.3–574.8)	<.001

FGF21: fibroblast growth factor 21; BMI: body mass index; SBP: systolic blood pressure; DBP: diastolic blood pressure; HDL: high-density lipid cholesterol; LDL: low-density lipid cholesterol.

Continuous variables are expressed as mean ± standard deviation or as median (interquartile range). Categorical variables are expressed as number (%).

^a^ Sixty-nine subjects taking anti-hypertensive medication were excluded from the analysis.

**Table 2.** Clinical characteristics of all 1051 participants according to FGF21 quartiles.

	Quartile 1	Quartile 2	Quartile 3	Quartile 4	*p* Value
*N*	262	264	263	262	
Age, years	41.7 ± 9.7	45.1 ± 10.4	46.7 ± 10.1	47.5 ± 9.7	<.001
Men, %	95 (36.3)	110(41.7)	134 (51.0)	141 (53.8)	<.001
BMI, kg/m^2^	22.3 ± 2.9	23.7 ± 3.7	24.4 ± 3.9	24.3 ± 3.3	<.001
SBP, mmHg^a^	112.9 ± 15.1	118.3 ± 15.2	121.0 ± 15.0	124.4 ± 15.4	<.001
DBP, mmHg^a^	74.7 ± 9.2	78.2 ± 9.8	79.6 ± 10.4	82.2 ± 11.1	<.001
Smoking, %	41 (15.6)	50 (18.9)	55 (20.9)	72 (27.5)	.008
Physical activity, %	122 (46.6)	127 (48.1)	108 (41.1)	132 (50.4)	.173
Alcohol consumption, %	10 (3.8)	15 (5.7)	24 (9.1)	24 (9.2)	.036
Hypercholesterolaemia, %	77 (29.4)	93 (35.2)	100 (38.0)	114 (43.5)	.008
Diabetes, %	10 (3.8)	18 (6.8)	29 (11.0)	27 (10.3)	.008
Hypertension, %	30 (11.5)	51 (19.3)	71 (27.0)	92 (35.1)	<.001
Anti-hypertensive medication, %	9 (3.4)	10 (3.8)	22 (9.4)	28 (10.7)	.001
Glucose, mmol/L	4.53 ± 1.35	4.67 ± 1.12	4.68 ± 0.98	4.75 ± 1.10	.143
Total cholesterol, mmol/L	4.69 ± 0.85)	4.93 ± 0.88	4.97 ± 0.89	5.08 ± 0.89	<.001
Triglycerides, mmol/L	1.01 ± 0.78	1.29 ± 0.84	1.61 ± 1.53	2.11 ± 2.06	<.001
HDL, mmol/L	1.45 ± 0.31	1.37 ± 0.29	1.35 ± 0.30	1.34 ± 0.28	<.001
LDL, mmol/L	2.55 ± 0.83	2.76 ± 0.66	2.79 ± 0.70	2.81 ± 0.71	<.001

FGF21: fibroblast growth factor 21; BMI: body mass index; SBP: systolic blood pressure; DBP: diastolic blood pressure; HDL high-density lipid cholesterol; LDL: low-density lipid cholesterol.

Continuous variables are expressed as mean ± standard deviation or as median (interquartile range). Categorical variables are expressed as number (%). Quartile 1: <156.6 pg/mL, quartile 2: 156.6–271.8 pg/mL, quartile 3: 271.9–429.1 pg/mL and quartile 4 ≥ 429.2 pg/mL.

^a^ Sixty-nine subjects taking anti-hypertensive medication were excluded from the analysis.

All analyses were performed in SPSS Version 27 (IBM Corporation, Armonk, NY), except for restricted cubic spline regression, which was performed in R (version 4.2.2; R Development Core Team; R Foundation for Statistical Computing in Vienna, Austria). Two-tailed *p* value equal to or less than .05 was considered statistically significant.

## Results

### Clinical characteristics

There were totally 1051 participants with a mean (SD) age of 45.2 (10.2) years included in this study. Two hundred and forty-four (23.2%) had hypertension and 480 (45.7%) were men. Table 1 shows the clinical characteristics of participants with and without hypertension. Compared to participants without hypertension, participants with hypertension were more likely to be older (*p* < .001), women (*p* < .001) and smokers (*p* = .004). Hypertensive patients had higher BMI (*p* < .001) and were likely to be diabetic (*p* = .015) compared to participants without hypertension. The medians of FGF21 level were 355.1 pg/mL (interquartile range 234.3–574.8 pg/mL) for hypertensive patients and 253.5 (interquartile range 136.9–403.3 pg/mL) for non-hypertensive participants.

Table 2 shows the clinical characteristics of participants according to FGF21 quartiles. In the overall population, the median FGF21 level was 271.8 pg/mL (interquartile range 156.6–429.2 pg/mL). Compared with participants that had lower serum FGF21, those that had higher serum FGF21 were more likely to be older (*p* < .001), men (*p* < .001) and smokers (*p* = .008). From quartile 1 to quartile 4, there is an increase in average age (from 41.7 to 47.5 years), average BMI (from 22.3 to 24.3 kg/m^2^), average systolic BP (from 112.9 to 124.4 mmHg) and average diastolic BP (from 74.7 to 82.2 mmHg), respectively (all *p* < .001). FGF21 level was significantly associated with hypertension (*p* < .001), hypercholesterolaemia (*p* = .008) and diabetes (*p* = .008).

### Association between FGF21 and BP

Table 3 shows the association between FGF21 and BP. Ln-transformed FGF21 level was associated with both systolic and diastolic BP (systolic BP: *B* = 4.45 [95% CI 3.41–5.49]; *p* < .001; diastolic BP: *B* = 2.72 [95% CI 2.03–3.42]; *p* < .001). After adjusting for sex and age, the association remained significant (systolic BP: *B* = 2.88 [95% CI 1.89–3.88]; *p* < .001; diastolic BP: *B* = 1.86 [95% CI 1.18–2.54]; *p* < .001). After adjusting for BMI, anti-hypertensive treatment, hypercholesterolaemia, diabetes, alcohol consumption, smoking and physical activity, the association still existed (systolic BP: *B* = 1.99 [95% CI 1.01–2.97]; *p* < .001; diastolic BP: *B* = 1.36 [95% CI 0.69–2.04]; *p* < .001). Ln-transformed FGF21 was also associated with both systolic and diastolic BP in men and women in the multivariable-adjusted model.

**Table 3.** Association of ln-transformed FGF21 level with blood pressure.

	Systolic blood pressure		Diastolic blood pressure	
	*B* (95% CI)	*p*	*B* (95% CI)	*p*
Overall				
Unadjusted model	4.45 (3.41–5.49)	<.001	2.72 (2.03–3.42)	<.001
Model 1	2.88 (1.89–3.88)	<.001	1.86 (1.18–2.54)	<.001
Model 2	1.99 (1.01–2.97)	<.001	1.36 (0.69–2.04)	<.001
Men				
Unadjusted model	2.08 (0.59–3.58)	.006	1.93 (0.89–2.96)	<.001
Model 1	2.00 (0.53–3.47)	.008	1.88 (0.85–2.90)	<.001
Model 2	1.48 (0.60–1.29)	.041	1.51 (0.51–2.52)	.003
Women				
Unadjusted model	5.34 (3.94–6.74)	<.001	2.47 (1.58–3.35)	<.001
Model 1	3.22 (1.85–4.59)	<.001	1.84 (0.92–2.76)	<.001
Model 2	2.22 (0.84–3.59)	.002	1.33 (0.39–2.26)	.006

Model 1: adjusted for sex (except in sex-specific analysis) and age. Model 2: further adjusted for BMI, anti-hypertensive treatment, hypercholesterolaemia, diabetes, alcohol consumption, smoking and physical activity.

**Figure 1.**
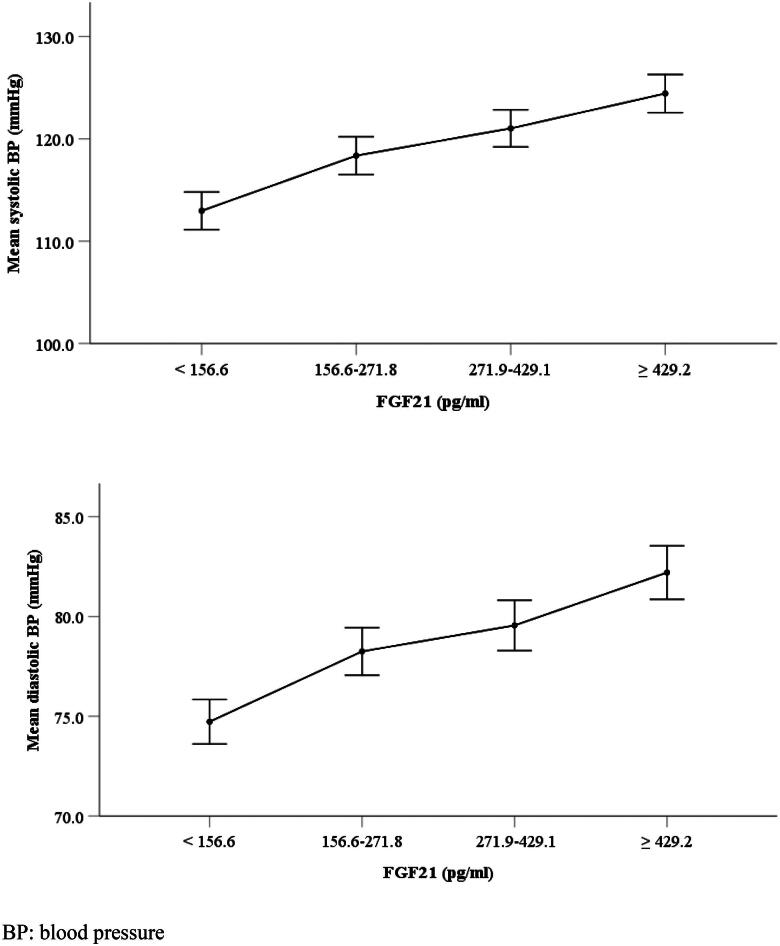
The linear relationship of FGF21 with systolic BP and diastolic BP. BP: blood pressure.

Figure 1 further shows the linear relationship of FGF21 with systolic and diastolic BP. Mean systolic and diastolic BP were gradually increased with FGF21 level groups from 112.9 to 124.4 mmHg and from 74.7 to 82.2 mmHg.

### Association between FGF21 and hypertension

Table 4 shows the association between FGF21 and hypertension. Higher serum FGF21 level was associated with higher risk of hypertension (quartile 4 vs. quartile 1, OR = 4.19 [95% CI 2.65–6.61]; *p* for trend <.001). After adjusting for sex and age, the association remained significant (quartile 4 vs. quartile 1, OR = 3.14 [95% CI 1.96–5.03]; *p* for trend <.001). After adjusting for BMI, hypercholesterolaemia, diabetes, alcohol, smoking and physical activity, the association still existed (quartile 4 vs. quartile 1, OR = 2.53 [95% CI 1.56–4.10]; *p* for trend <.001). Increased ln-transformed FGF21 level was associated with increased risk of hypertension after the adjustment for sex, age, BMI, hypercholesterolaemia, diabetes, alcohol, smoking and physical activity (OR = 1.42 [95% CI 1.18–1.71]; *p* <.001, Table S1).

**Table 4.** The association between FGF21 quartiles and hypertension.

Overall population	Odds ratio (95% confidence interval)		
	Unadjusted model	Model 1	Model 2
Quartile 1	1.00 (ref.)	1.00 (ref.)	1.00 (ref.)
Quartile 2	1.85 (1.14–3.02)*	1.58 (0.96–2.62)	1.29 (0.77–2.16)
Quartile 3	2.86 (1.79–4.57)***	2.21 (1.36–3.58)**	1.63 (1.00–2.73)
Quartile 4	4.19 (2.65–6.61)***	3.14 (1.96–5.03)***	2.53 (1.56–4.10)***
*p* for trend	<.001	<.001	<.001

^*^ *p* < .05, ** *p* < .01, *** *p* < .001.

Quartile 1: <156.6 pg/mL, quartile 2: 156.6–271.8 pg/mL, quartile 3: 271.9–429.1 pg/mL and quartile 4 ≥ 429.2 pg/mL. Model 1: adjusted for sex and age. Model 2: further adjusted for BMI, hypercholesterolaemia, diabetes, alcohol, smoking and physical activity.

In Figure S1, we used restricted cubic splines to visualise the relation between FGF21 and hypertension. The plot shows a slight decrease in odd ratio of hypertension within the lower range of ln-transformed FGF21. A marked change in the odds ratio of serum FGF21 at hypertension risk occurred in the 113.3–544.6 pg/mL range (ln-transformed FGF21, 4.7–6.3; estimate from the spline). The odds ratio of hypertension increased slowly but steadily among those with serum FGF21 above 544.6 pg/mL.

### Sensitivity analysis

In the sensitivity analysis, ln-transformed FGF21 was associated with hypertension after excluding participants who had hypertensive medication (Table S2). After adding eGFR in the multivariable-adjusted model, ln-transformed FGF21 remained significantly associated with both systolic and diastolic BP (Table S3).

## Discussion

In this population-based study, circulating FGF21 was associated with both systolic and diastolic BP. The association persisted after adjusting for sex, age, BMI, hypercholesterolaemia, diabetes, alcohol consumption, smoking and anti-hypertensive treatment. A robust association between FGF21 and hypertension was also found in the fully adjusted model. In addition, serum FGF21 level positively correlated with age, BMI, diabetes and lipid profiles including total cholesterol, triglycerides, high-density lipid cholesterol and low-density lipid cholesterol.

To our knowledge from literature review, this is the first study to elucidate the relationship of FGF21 with BP in the Asian population. Our findings suggested that FGF21 could be a biomarker that can be measured in the onset and development of hypertension according to our up-to-date data on a Chinese population. A cross-sectional study conducted in US adults showed that serum FGF21 level, independent of age, BMI, eGFR and blood glucose, was associated with hypertension [21]. However, the prevalence of hypertension is discrepant between Western and Eastern countries. A recent study from the Non-Communicable Disease Risk Factor Collaboration reported that the prevalence of hypertension is higher in central and eastern Europe (men: 42.6–55.9%, women: 34.0–46.9%) compared with that in China (men: 30.2%; women: 24.1%) [13]. In the Multi-Ethnic Study of Atherosclerosis, also known as MESA, average level of angiotensinogen and prevalence of hypertension differed significantly among racial/ethnic groups (White, Chinese, Black and Hispanic) [22]. The heterogeneity of hypertension among different ethnic groups is related to the variability in sodium retention [23], BMI [24] and socioeconomic factors [25]. Also, circulating FGF21 level was strongly associated with adiposity [11]. Body fat percentage and lean mass differed among ethnic groups [12]. Sex differences in circulating FGF21 level has been found in a Danish study [26]. Given the hypertension and circulating FGF21 differences between Western and Eastern populations, it is logical to deduce that the relationship between circulating FGF21 and hypertension in Asian population could be different from Western population. Our study fills a void and confirms the association between FGF21 and hypertension in Asian population.

Prospective studies have reported that FGF21 is a biomarker of predictive value rather than a risk factor for diseases. Very recently, the PREVEND study has suggested that FGF21 was a promising biomarker for T2D [27]. In the study, 5244 participants were included at baseline, among whom 299 developed T2D during a follow-up period of 7.3 years. Time-to-event analysis showed that there was a significant association between FGF21 and T2D (HR per doubling: 1.26 [95% CI 1.06–1.51]) in participants without impaired fasting glucose instead of those who had impaired fasting glucose, which indicated that FGF21 was an early biomarker of T2D before the development of impaired fasting glucose [27]. In a study including multi-ethnic populations (non-Hispanic white, African American, Hispanic American and Chinese American), baseline characteristics showed that elevated FGF21 level was associated with the prevalence of metabolic syndrome. Among participants who did not have the metabolic syndrome at baseline, higher FGF21 level was associated with the development of metabolic syndrome (quartile 4 vs. quartile 1, HR = 1.76 [95% CI 1.46–2.12]) [28]. A meta-analysis revealed that there was a significant association between FGF21 level and coronary artery disease (HR 1.29 [95% CI 1.06–1.55]) [29]. Moreover, FGF21 was a predictor for all-cause mortality and cardiovascular mortality [29]. In the Multi-Ethnic Study of Atherosclerosis, 820 of 5768 participants developed incident cardiovascular disease after a follow-up period of 14 years. FGF21 was significantly associated with cardiovascular disease (HR 1.12 [95% CI 1.03–1.12]) [30]. These studies provided evidence for the association of FGF21 with T2D, metabolic syndrome, cardiovascular diseases and all-cause mortality.

FGF21, as a potential biomarker, plays an important role in the modulation of BP. The possible mechanisms have been previously reported. FGF21 induces angiotensin-converting enzyme 2, which mediates the transformation from angiotensin II to angiotensin-(1–7). The activation of the ACE2/angiotensin-(1–7) axis results in the improvement of BP and vascular dysfunction [31]. Besides, FGF21 has anti-inflammatory and anti-fibrotic effects. Administration of FGF21 reduces the expression of inflammatory factors and fibrosis-related factors, thereby suppressing cardiac inflammation and fibrosis, which may lead to a reduction in BP [32]. Moreover, adipose tissue provides mechanical protection for blood vessels and plays a role in the regulation of vascular tone [33]. FGF21 derived from brown adipose tissue has been found to lower BP and protect against hypertension [34]. Another mechanism through which FGF21 influences BP is by inducing the production of adiponectin. FGF21 deficiency results in severe hypercholesterolaemia. After replenishing FGF21, the adipocyte production of adiponectin increases, leading to a reduction in lipids and BP [16]. FGF21 has also been reported to have a direct effect on hypothalamic neurons, which stimulate the hypothalamic-pituitary-adrenal axis to produce corticosterone [35] and regulate BP. These findings provide a pathophysiological basis for the association of FGF21 with BP.

The association of FGF21 with BP and hypertension in our study supports the concept of FGF21 resistance. FGF21 is involved in the regulation of BP and contributes to its reduction. However, circulating level of FGF21 is markedly elevated in those with hypertension. The paradoxical changes of FGF21 level have also been shown in previous studies. FGF21 has been found to stimulate glycogen production in liver and induce lipolysis in white adipose tissues during fasting and starvation. Surprisingly, instead of having deficient levels, obese diabetic db/db mice and obese individuals exhibit increased levels of FGF21 [6]. FGF21 resistance is promoted by central resistin, via toll-like receptor 4, through the downregulation of FGF21 hypothalamic expression and both hypothalamic and peripheral expression of its receptor components [36].

FGF21 is also a promising therapeutic target for hypertension. LY2405319, an analogue of FGF21, has been tested in obese patients with T2D in a randomized, placebo-controlled, double-blind proof-of-concept trial [37]. At the end of the 28-day treatment period, the usage of FGF21 analogue produced favourable effects on circulating lipid profiles (triglycerides, low-density lipoprotein cholesterol and high-density lipoprotein cholesterol) and contributed to improvements in dyslipidaemia. Beneficial effects on body weight, fasting glucose and fasting insulin were also observed [37]. Obesity and dyslipidaemia are key risk factors for hypertension [38]. Our previous study has proposed the common pathways between diabetes and hypertension, including obesity, inflammation, oxidative stress and insulin resistance [39]. Given the important role of diabetes and dyslipidaemia in the development of hypertension, we speculate that FGF21 is also a therapeutic target for hypertension. Although the use of the FGF21 analogue has not been applied in patients with hypertension, based on the mechanisms between FGF21 and BP, FGF21 is a promising therapeutic target for treating hypertension.

This study has several strengths. The participants in this study come from communities in Shenzhen, a city known for its migrant population. As a result, the study sample may offer a representation of the population in China, at least in terms of ethnic origin and genetics. The relatively large sample size of our study allowed us to perform subgroup analysis with a substantial number of participants. There is a limitation in our study. Due to the nature of cross-sectional study, it inevitably avoids determining the causal relationship between FGF21 and hypertension. Based on the findings from our preclinical study [16] and possible underlying mechanisms, it is reasonable to understand that FGF21 is a biomarker for hypertension in this study, which is conducted in human beings.

## Conclusions

In conclusion, FGF21 is associated with BP and hypertension. FGF21 is a potential biomarker for the assessment of hypertension. Further studies investigating its role in hypertension may reveal new drug targets.

## Supplementary Material

Supplemental Material

## Data Availability

The data that support the findings of this study are available from the corresponding author upon reasonable request.
