# Supplementary material for: Circulating fibroblast growth factor 21 is associated with blood pressure in the Chinese population: a community-based study
Source: Ann Med. 2025 May 12;57(1):2500689. doi: 10.1080/07853890.2025.2500689 (PMC12077425; doi:10.1080/07853890.2025.2500689)
Supplement: Supplemental Material [file IANN_A_2500689_SM1667.zip › supplementary_tables.docx]

Table S1. ORs (95% CIs) of hypertension when serum FGF21 level used as continuous variables.

|  | OR | 95%CI | P |
| --- | --- | --- | --- |
| Overall (N=1051) |  |  |  |
| Unadjusted | 1.71 | 1.44-2.04 | <0.001 |
| Model 1 | 1.54 | 1.29-1.84 | <0.001 |
| Model 2 | 1.42 | 1.18-1.71 | <0.001 |
|  |  |  |  |
| Men (N=480) |  |  |  |
| Unadjusted | 1.41 | 1.12-1.77 | 0.004 |
| Model 1 | 1.41 | 1.12-1.78 | 0.004 |
| Model 2 | 1.35 | 1.06-1.73 | 0.016 |
|  |  |  |  |
| Women (N=571) |  |  |  |
| Unadjusted | 2.00 | 1.52-2.62 | <0.001 |
| Model 1 | 1.68 | 1.26-2.23 | <0.001 |
| Model 2 | 1.51 | 1.13-2.03 | 0.006 |

Model 1: adjusted for sex and age

Model 2: further adjusted for BMI, hypercholesterolaemia, diabetes, alcohol, smoking and physical activity

Table S2. Sensitivity analysis by excluding participants who had hypertensive medication

|  | OR | 95%CI | P |
| --- | --- | --- | --- |
| Overall |  |  |  |
| Unadjusted | 1.66 | 1.37-2.01 | <0.001 |
| Model 1 | 1.52 | 1.25-1.85 | <0.001 |
| Model 2 | 1.44 | 1.17-1.76 | <0.001 |
|  |  |  |  |
| Men |  |  |  |
| Unadjusted | 1.43 | 1.11-1.86 | 0.006 |
| Model 1 | 1.43 | 1.11-1.85 | 0.007 |
| Model 2 | 1.43 | 1.09-1.87 | 0.010 |
|  |  |  |  |
| Women |  |  |  |
| Unadjusted | 1.82 | 1.35-2.45 | <0.001 |
| Model 1 | 1.60 | 1.17-2.19 | 0.003 |
| Model 2 | 1.48 | 1.07-2.04 | 0.017 |

Model 1: adjusted for sex and age

Model 2: further adjusted for BMI, hypercholesterolaemia, diabetes, alcohol, smoking and physical activity

Table S3. Sensitivity analysis by adjusting for estimated glomerular filtration rate

|  | Systolic Blood Pressure | | Diastolic Blood Pressure | |
| --- | --- | --- | --- | --- |
|  | B (95% CI) | P | B (95% CI) | P |
| Overall |  |  |  |  |
| Unadjusted | 4.45 (3.41-5.49) | <0.001 | 2.72 (2.03-3.42) | <0.001 |
| Model 1 | 2.88 (1.89-3.88) | <0.001 | 1.86 (1.18-2.54) | <0.001 |
| Model 2 | 2.03 (1.03-3.03) | <0.001 | 1.40 (0.71-2.09) | <0.001 |

Model 1: adjusted for sex and age

Model 2: further adjusted for BMI, hypercholesterolaemia, diabetes, alcohol, smoking, physical activity and estimated glomerular filtration rate
