# Supplementary figures and images for: Circulating fibroblast growth factor 21 is associated with blood pressure in the Chinese population: a community-based study
Source: Ann Med. 2025 May 12;57(1):2500689. doi: 10.1080/07853890.2025.2500689 (PMC12077425; doi:10.1080/07853890.2025.2500689)

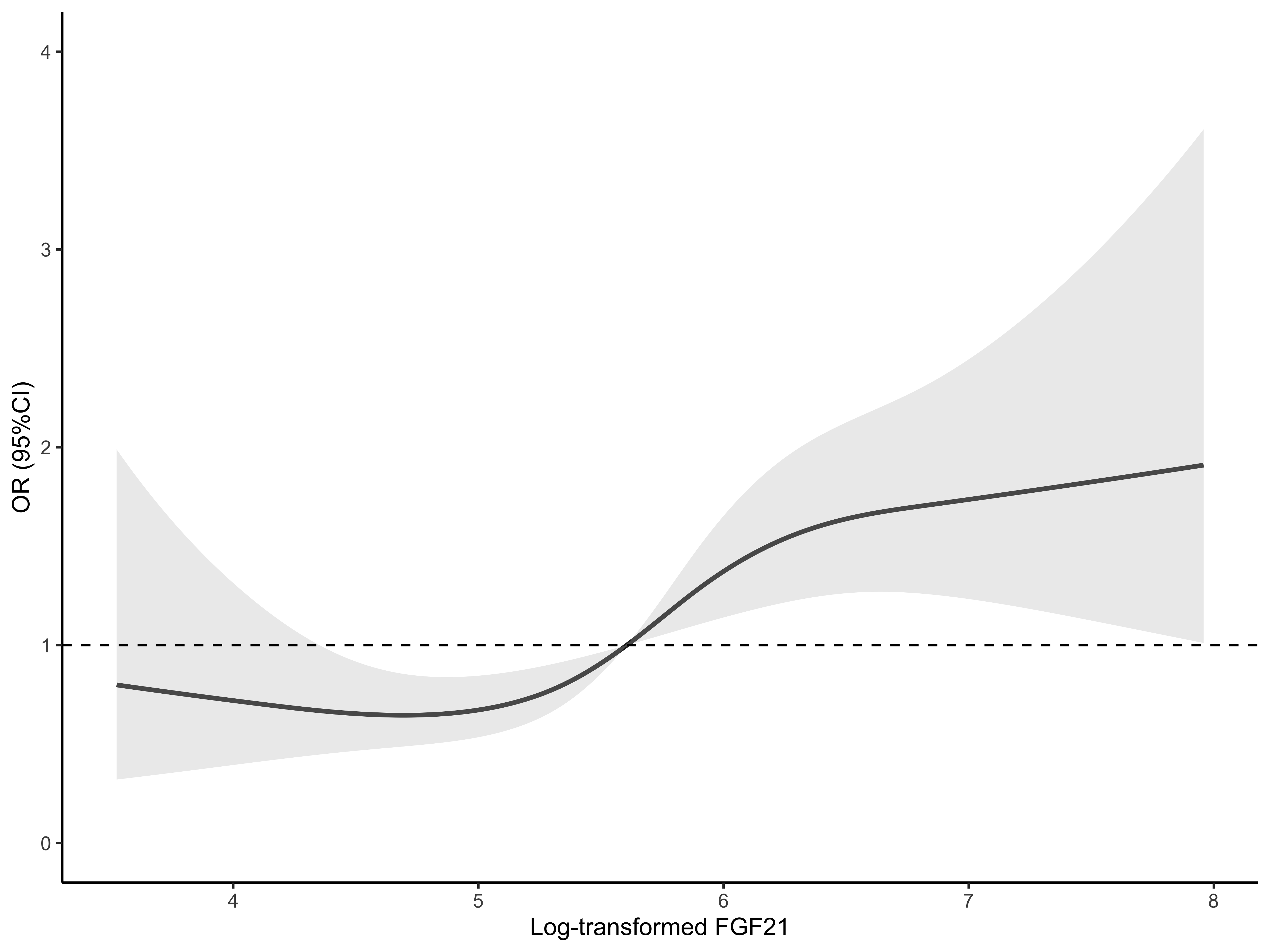

Supplement: Supplemental Material [file IANN_A_2500689_SM1667.zip › Figure_S1_high.png]
